# Effects of a Change from an Indoor-Based Total Mixed Ration to a Rotational Pasture System Combined with a Moderate Concentrate Feed Supply on Immunological Cell and Blood Parameters of Dairy Cows

**DOI:** 10.3390/vetsci6020047

**Published:** 2019-05-24

**Authors:** Julia Hartwiger, Melanie Schären, Jana Frahm, Susanne Kersten, Liane Hüther, Helga Sauerwein, Ulrich Meyer, Gerhard Breves, Sven Dänicke

**Affiliations:** 1 Institute of Animal Nutrition, Friedrich-Loeffler-Institut (FLI), Federal Research Institute for Animal Health, Bundesallee 37, 38116 Braunschweig, Germany; juliahartwiger@gmx.de (J.H.); Melanie.Schaeren@uni-leipzig.de (M.S.); Susanne.Kersten@fli.de (S.K.); Liane.Huether@fli.de (L.H.); Ulrich.Meyer@fli.de (U.M.); Sven.Daenicke@fli.de (S.D.); 2 Institute for Animal Science Physiology & Hygiene, University of Bonn, Katzenburgweg 7–9, 53115 Bonn, Germany; sauerwein@uni-bonn.de; 3 Institute of Physiology, University of Veterinary Medicine Hannover, Foundation, Bischofsholer Damm 15, 30173 Hannover, Germany; Gerhard.Breves@tiho-hannover.de

**Keywords:** dairy cows, ration change, pasture, confinement, immune system, inflammatory markers, nutrient

## Abstract

In spring, transition from a total mixed ration (TMR) to a full grazing ration with moderate concentrate supply influences cow’s metabolism. It has been shown that feeding moderate amounts of concentrate during fulltime grazing did not prevent energy shortage and lipomobilization, alterations in energy metabolism, decreasing milk production and loss in body weight. As diet change and energy balance are closely related to immune reactivity, in this trial the effect of transition to pasture on specific immune parameters of cows was documented. Over a 12-week trial 43 dairy cows were observed during transition from confinement to pasture (PG; *n* = 22) and compared to cows fed TMR indoor (CG; *n* = 21). The CG stayed on a TMR based ration (35% corn silage, 35% grass silage, 30% concentrate; dry matter (DM) basis), whereas the PG slowly switched to a pasture -based ration (week 0 and 1 = TMR, week 2 = TMR and 3 h pasture·day^−1^, week 3 and 4 = TMR and 12 h pasture·day^−1^, and week 5 to 11 = pasture combined with 4.5 kg DM concentrate·cow^−1^·day^−1^). Inflammatory markers like blood haptoglobin or tryptophan to kynurenine ratio did not indicate acute phase reaction. Proportions of CD4^+^ (T-helper cells) and CD8^+^ cells (cytotoxic T-cells) remained uninfluenced as well. White blood cell concentration and its subpopulation of granulocytes increased over time in the PG. Stimulation ability of peripheral blood mononuclear cells to mount an oxidative burst significantly increased during the trial, too. The endogenous antioxidant state as characterized by glutathione peroxidase (GPx) and superoxide dismutase (SOD) activity in blood of the PG did not change, whereas the vitamin E concentration reached the highest level at the end of the trial. The 25-CHO metabolites of vitamin D increased as soon as the PG had pasture access, whereas the other metabolite 25-ERG decreased. The results of this study indicate that transition to pasture affects immune related parameters. However, the consequences of the observed effects on health status of the pasture group need to be clarified in further studies with a defined concurrent immune challenge.

## 1. Introduction

Grazing cattle are a symbol of a welfare friendly system, as it allows the expression of a normal behavior which may be restricted in confinement. Vernal transition from a total mixed ration (**TMR**) indoor system to a full-grazing system combined with small and moderate amounts of concentrate supply was shown to result in an energy deficiency in high yielding Holstein cows [1,2,3,4,5]. During this period the rumen was also markedly challenged due to diet and behavior changes with alterations in rumen microbiota, fermentation profile, daily pH course and changes of rumen papillae size [1,2,3,4,5]. All the observed changes during transition lead to complex physiological and structural adaptions of the rumen. These physiological effects with consequences on health and performance can be preconditions for changes in immune reactions, similar to those observed during the periparturient period [6].

The immune system consists of the innate and the adaptive part, which are managed by different cell populations and molecule types. The major aim of the immune defense is the protection of the host against diseases triggered by pathogens in order to keep the animal in a good state of health. In confinement as well as in pasture systems, responses to pathogen challenges can have impacts on performance and animal welfare. Changing stressors either of nutritional, environmental, microbiotic or parasitic origin can have effects on the immune response [7,8]. 

Possible influences of diet and other environmental factors on immune system functions in transition cows were discussed by Dänicke et al. [9]. Although the transition from late gestation to early lactation is physiologically different from the transition of a cow from confinement to pasture, there are some common metabolic features. 

A change of the chemical composition of the diet as examined in the study of Dänicke et al. [9] also occurs during transition from confinement with TMR feeding to pasture grazing [1]. The chemical composition of pasture differs greatly compared to TMR. Pasture contains large amounts of high fermentable carbohydrates, protein and has low dry matter (**DM**) content. Especially the low DM content and the grazing itself reduce dry matter intake (**DMI**). As shown in our study [1] and a previous one [3], the increase in physical activity due to walking and grazing leads to higher energy requirements which are difficult to meet by the animals during transition to pasture and up to 6 weeks afterwards. Due to of the lower DMI compared to confinement housing there is a gap between nutrient requirement and supply, resulting in a negative energy balance. That is why cows experience an energy deficit eventually accompanied by increased beta-hydroxy-butyrate (**BHB**) and non-esterified-fatty acid concentration (**NEFA**; [1,3]) in blood serum due to triggered lipolysis. The observed changes in energy related blood parameters have been associated with immune system function [9,10]. During energy deficiency the immune activity requires energy as well. For this reason, it might be concluded that the energy status of the pasture group during transition may have influenced on immune system function and therefore health traits [11]. On the other hand, spring grass combined with concentrate supply can lead to subacute ruminal acidosis during the first weeks on fulltime grazing [4]. This could lead to inflammation of the papillae and thus to the activation of an immune response. Trevisi et al. [12,13] hypothesized in their study that forestomachs can sense sterile and infectious stressors and react to them, and that there and elsewhere in the body immune response can occur in case of local inflammation or inflammatory conditions, too. 

Catabolic pathways, especially at the cellular level with high NEFA concentration (2 mM) can lead to an imbalance between oxidant and antioxidant variables, influencing viability and generation of reactive oxygen species (**ROS**) of polymorphonuclear leucocytes (**PMN**; [11]). However, metabolic adaptations to counterbalance the energy deficit through lipid mobilization resulting in elevated lipid accumulation in liver or decreasing rumen pH due to the influence of high fermentable grass [4] can trigger inflammatory responses [14] as indicated by an increase of acute phase proteins in blood such as haptoglobin [15] or other inflammatory parameters like the ratio between tryptophan and its catabolized metabolite kynurenine (**Kyn**; [16]). It is well known that changes in nutrition and season can influence inflammatory markers [17] and oxidative stress (**OS**) biomarkers [18] as well as vitamin status [19,20] of ruminants. 

Studies investigating immune modulations during the transition from confinement to pasture are limited. Due to the lack of knowledge, it is difficult to evaluate the impact of this time period on general health aspects. Therefore, the present study aims to evaluate possible effects of transition from an TMR indoor-based feeding to a full grazing ration with supplementation of 4.5 kg DM concentrate·cow^−1^·day^−1^ on different parameters of the immune system in lactating dairy cows.

## 2. Materials and Methods 

Experimental work was carried out at the experimental station of the Institute of Animal Nutrition, Friedrich-Loeffler-Institut (FLI) in Braunschweig, Germany. The experiment was performed in compliance with the German legislation on animal protection (Animal Welfare Act) and approved by the Lower Saxony State Office for Consumer Protection and Food Safety (LAVES, Oldenburg, Germany, file number 33.19-42502-04-15/1858) in consultation with an independent ethics committee and was supported by the Ministry for Science and Culture Section of Lower Saxony (MWK), Hannover, Germany.

### 2.1. Experimental Design and Treatments

The experimental design, treatments, DMI, rations, climate data, animal performance, energy metabolism, physical activity, clinical chemistry and erythrogram were previously described [1,2] and were based on the setup described in [3,4], except for the grazing system (rotational vs. stationary) and the amount of concentrate supplementation (1.75 vs. 4.5 kg DM concentrate·cow^−1^·day^−1^).

Briefly, the experimental work started on April 18, lasted about 12 weeks, and ended on July 8, 2016. The present investigations included 43 German Holstein cows (parity: 2.2 ± 1.4; 164 ± 33 days in milk; 27.6 ± 2.1 kg milk·cow^−1^·day^−1^; at the beginning of the trial) which were either assigned to a pasture (**PG**; *n* = 22) or a confinement group (**CG**; *n* = 21). The animals were equally divided according to their parity, DMI and performance. 

The CG received a TMR (35% maize silage, 35% grass silage, 30% concentrate, DM-basis) throughout the trial, whereas the PG gradually transitioned towards pasture feeding (weeks 0 and 1: TMR-only during confinement, week 2: TMR during confinement and 3 h·day^−1^ on pasture, weeks 3 and 4: TMR during confinement and 12 h·day^−1^ on pasture, week 5 to 11: pasture-only plus 4.5 kg DM concentrate·cow^−1^·day^−1^). The offered concentrate contained a mineral mix, which included 100,000 IU of 25-hydroxycholecalciferol (**25CHO**, a metabolite of vitamin D) and 1500 mg of vitamin E per kg mineral feed. The concentrate of the TMR of both groups included 1.5% of the mineral mix and the concentrate of the PG included 9% of the mineral mix during all day grazing (week 5 to 11). During confinement housing both groups received about 149 mg vitamin E cow^−1^·day^−1^ through concentrate supplementation. During fulltime grazing (week 5 to 11) the PG received 608 mg vitamin E·cow^−1^·day^−1^ through concentrate supplementation. Between week 5 and 11, animals spent 2 h daily in confinement for milking and concentrate supply.

A rotational grazing system dominated by perennial ryegrass (paddock size: 1.6 ± 0.3 ha each) was performed. On average all four paddocks were covered to 79.5% ± 13.6% with grass, 7.5% ± 6.9% with herbs and 4.4% ± 7.4% with legumes (estimated pasture coverage; mean ± SD). All cows entered a paddock when sward height had reached an average of 14 cm and left the paddock when the sward height was around 8 cm, or when the visual assessment of the pasture indicated an insufficient composition. Paddock sward height was checked daily with RPM F400, (Farmworks Systems Ltd., Manawatu-Wanganui, New Zealand). The average pasture allowance amounted to 142 ± 46 kg of DM grass·cow^−1^·day^−1^ in the beginning of each grazing period and decreased on average to 87 ± 18 kg of DM grass·cow^−1^·day^−1^ after 6 days of grazing.

The chemical composition of the pasture was documented weekly from week 2 on. For this, we took pooled samples over one week with the help of an electronic scissor in areas were the cows spent most of the time for grazing and exclusively from the upper half of the plant. The composition of the TMR was analyzed two times. All chemical methods concerning feed analyses are based on the procedures recommended by VDLUFA [21], as described in detail by Schären, et al. [3]. Briefly, the average chemical composition of the TMR of both groups and pasture is described in Table 1.

In the CG individual DMI and water intake was recorded automatically using electronic weighing troughs with ear tag detection (computerized feeding station Insentec Typ RIC, B. CF., Markenesse, The Netherlands). DMI on pasture was recorded in week 6 and 7 using the n-alkane marker method and calculated in week 2 to 11, based on modified equations according to Heublein et al. [22]. For a more detailed description of DMI and its calculation on pasture, please look up Hartwiger et. al. [1].

Body weight was measured twice daily, and body condition score was documented once per week. Milk yield was measured twice daily, and milk samples were taken twice per week for analysis. 

Weather and barn climate condition were recorded daily. The data were used to calculate the daily temperature humidity index (THI). The outdoor THI average was 58.8 ± 6.1 being 3.9 ± 1.9 (mean ± SD) units lower compared to indoors. Periods of a mild heat stress (THI between 65 and 70 [23,24,25]) were outdoors in weeks 6 and 9 and indoors in weeks 6, 7, 9 and 11.

Further information about metabolic changes and adaption of different rumen variables concerning this trial were recently described in detail elsewhere [1,2].

### 2.2. Animal Measurements

#### 2.2.1. Blood Sampling

Blood samples of 43 animals (PG: 22, CG: 21) were collected weekly by puncturing a vena jugularis externa after morning milking for hematology. At three time points during the trial (week 0, 6, 11) blood samples for serum metabolites and flow cytometry were collected. The samples were always taken on Mondays and thus reflect the health status of the previous week. EDTA blood samples were directly used for hematology and flow cytometry, while heparinized plasma and serum samples were centrifuged at 2123× *g* for 15 min at 15 °C (Heareus Verfuge^®^ 3.0R Heareus, Osterode, Germany) and frozen in aliquots either at −20 or −80 °C until further analysis.

#### 2.2.2. Hematology

A blood cell count was performed (Celltac α MEK-6450, Nihon Kohden Corporation, Tokyo, Japan) using EDTA blood samples; measured parameters included hemoglobin and differential white blood cell counts into lymphocytes, monocytes, granulocyte subtypes including neutrophils and eosinophils.

#### 2.2.3. Serum Metabolites

Serum concentrations of **vitamin E (α-Tocopherol) and vitamin D** measured via its metabolites 25-hydroxyergocalciferol (25ERG) and 25-hydroxycholecalciferol (**25CHO**) were determined by high-performance liquid chromatography with diode array detection (HPLC-DAD; Shimadzu, Kyoto, Japan). For the measurement of vitamin D metabolites, first a solid phase extraction (Phree^TM^ Phospholipid Removal 1 mL tube, Phenomenex) with acetonitrile/methanol was performed. After that the combined extracts were evaporated at 40 °C under nitrogen-flow, the dried residues were dissolved in methanol/distilled water (40/60) and filtered (syringe filters, 13 mm, 0.45 µm, PVDF, amchro GmbH) before HPLC injection. The HPLC parameters were as follows: oven temperature: 40 °C, autosampler temperature: 4 °C, column: Synergi 4 µm Hydro-RP 80 A; 250 × 3.0 mm, gradient elution with acetonitrile (mobile phase A) and methanol/distilled water (40/60) (mobile phase B), DAD at 265 nm. For vitamin E analysis [26,27], serum samples were diluted with a mixture of ice-cold ethanol containing 0.01% butylated hydroxytoluene for protein precipitation. After cooling on ice for 10 min, samples were mixed with n-hexane for fat and vitamin E extraction, followed by centrifugation at 20,800× *g* for 15 min at 4 °C (Eppendorf centrifuge^®^ 5417R, Eppendorf, Hamburg, Germany). The extraction was performed three times. The upper hexane layers were collected in an amber flask and evaporated in a nitrogen stream at 40 °C. The residue was dissolved in mobile phase and after filtration (syringe filters, 0.45 μm, PVDF, amchro GmbH) 20 µL were injected into the HPLC. HPLC-measurement was carried out under the following conditions: oven temperature: 25 °C, autosampler temperature: 4 °C, column: Intersil 150 Å; ODS-2; 5 µm; 150 × 3.0 mm, mobile phase: isocratic methanol, DAD: 295 nm.

The haptoglobin concentrations in serum were analyzed in duplicate using an in-house developed ELISA as described previously by Hiss et al. [28,29]. A bovine serum calibrated against a standard obtained from an EU COST action on the standardization of animal acute phase proteins (QLK5-CT-1999-0153; Skinner [30]) was used to generate the calibration curve. In brief, the assay was performed as follows: microtiterplates were first coated with bovine serum, then blocked with casein and stored at 4 °C. Before use, the plates were decanted and then the polyclonal antiserum against haptoglobin (generated in rabbits against haptoglobin purified from bovine serum) was added and incubated together with the samples, standard or controls for 2 h at room temperature. After three times washing, the second enzyme-labeled antibody (monoclonal mouse anti-rabbit IgG (γ-chain specific)-peroxidase; A1949, Sigma-Aldrich) was added. After incubation and further washings, tetramethylbenzidin was used as chromogene, and the optical density was determined at 450 nm with a microtiter plate reader (Synergy H1 Reader, BioTek, ELX800, BioTec Instruments, Inc., Winooski, VT, USA). The haptoglobin concentration was calculated from the calibration curve. Our cut-off threshold for variation was 15%; the coefficients of intra- and inter-assay variation were determined from the control samples assayed on each plate and were 9.25% and 9.19%, respectively.

The tryptophan (**Trp**) and kynurenine (**Kyn**) concentrations were analyzed in serum samples, as described in detail in Hüther et al. [31]. Briefly, serum samples were mixed with ice cold ethanol for protein precipitation and centrifuged, followed by three washing steps with hexane for fat extraction. After evaporation of the aqueous phase in a nitrogen stream, the residue was dissolved in a mobile phase A followed by filtration and injection into the HPLC system (Shimadzu, Kyoto, Japan). Separation of both metabolites was performed using a reversed phase C18-column (Inertsil ODS-2, 150 × 3.0 mm i.d., 5 μm particle size, Agilent; Böblingen, Germany) and gradient elution. The mobile phase A consisted of 10 mM sodium 1-hexanesulfonate monohydrate, 0.5% (*v*/*v*) o-phosphoric acid and 0.5% (*v*/*v*) acetonitrile in ultrapure water; the mobile phase B consisted of 100% acetonitrile. For detection a DAD (Trp: 278 nm, Kyn: 360 nm) was used. The Kyn/Trp ratio was calculated based on their molar concentrations in serum.

#### 2.2.4. Flow Cytometry

The ability of granulocytes to generate ROS and to elicit oxidative burst was determined by flow cytometry (FACS Canto II, BD Biosciences, San Jose, CA, USA) using a Dihydrorhodamine (**DHR**) assay. The method is based on the capacity of polymorphonuclear leukocytes (**PMN**) to produce ROS, either under unstimulated conditions (basal) or after stimulation with Tetradecanoyl-12, 13-Phorbol acetate (**TPA**) which activates the NADPH-oxidase and induces an oxidative burst. The non-fluorescent DHR^+^123 is an uncharged ROS indicator that can passively diffuse across cell membranes where it is oxidized to fluorescent Rhodamine123 (R123). Briefly, whole blood samples were incubated with either DHR (40 μM, Molecular Probes Inc., Eugene, OR, USA) or DHR and TPA (2000 nM, Sigma-Aldrich, Steinheim, Germany) for 15 min at 37 °C. After 10 min lysis of erythrocytes and fixation using lysis buffer (BD Pharm Lyse^TM^, BD Biosciences, San Jose, CA, USA) samples were washed with HEPES-buffered saline (HBS) and measured in duplicates by flow cytometer. By performing forward and side scatter measurements the PMNs were gated according to their size and granularity. At least 10,000 granulocytes were examined. Basal ROS^+^PMN (%) shows the proportion of ROS producing PMN. TPA stimulated ROS^+^PMN (%) shows the proportion of cells capable of ROS production upon stimulation. The mean fluorescence intensity (**MFI**) quantifies the amount of ROS formation per cell (basal and stimulated with TPA). Based on Dänicke et al. [32], total counts of basal ROS producing peripheral blood granulocytes was calculated (G·L^−1^; total blood granulocytes multiplied with the proportion of basal ROS^+^PMN).

T-cell phenotyping was performed in whole blood samples quantifying specific cluster of differentiation (**CD**). EDTA blood was double stained with monoclonal antibodies against CD4 (mouse anti bovine CD4: FITC, BioRad, Hercules, CA, USA) and CD8 (mouse anti bovine CD8: PE, BioRad, Hercules, CA, USA) or the corresponding isotype controls (mouse IgG2a negative control: RPE and mouse IgG2b: FITC negative control, BioRad, Hercules, CA, USA). After 30 min incubation at room temperature, red blood cells were lysed with lysis buffer (BD Bioscience, San Jose, CA, USA) for 10 min at room temperature. Samples were centrifuged, resuspended in HBS and measured by using FACS Canto II (BD Biosciences, San Jose, CA, USA). Finally, a CD4^+^ and CD8^+^ T-cell screening followed, by setting an acquisition gate for the lymphocyte population based on their side and forward scattering properties. Not less than 10,000 lymphocytes were counted and stored in list mode data files. The spillover of both fluorochromes (FITC, PE) was compensated using the BD FACS Diva^TM^ Software (BD Biosciences, San Jose, CA, USA). Results were reported as percentage of CD4^+^ and CD8^+^ cells of the total lymphocytes and as the ratio between both cell subsets.

#### 2.2.5. Enzyme Assays 

Serum antioxidant enzyme activity of glutathione peroxidase (**GPx**) and superoxide dismutase (**SOD**) was measured and referred to hemoglobin in red blood cell lysate. GPx activity was measured with Ransel glutathione peroxidase assay (Randox Laboratories, Crumlin, UK) according to the manufacturer’s protocol, based on the method of Paglia and Valentine [33]. SOD capacity was measured with Ransel superoxide dismutase assay reagents (Randox Laboratories, Crumlin, UK) according to the manufacturer’s protocol.

### 2.3. Statistical Analyses

Statistical analyses were performed using the Software SAS Enterprise Guide 7.1 (SAS Institute Inc., Cary, NC, USA). The presented data were evaluated by the same procedures as the parameters discussed in Hartwiger et al. [1,2] to enable a complex time dependent evaluation and to discuss the results as group-specific LS-means. For this reason, normal distribution of the present analyzed data was assumed. Repeated measurements were analyzed with the procedure mixed using a restricted maximum likelihood model (REML). In this mixed procedure, cow, time and diet group were defined as class statements. Time (= sampling weeks), diet group (PG or CG) and their interaction were defined as fixed factors in the model. Time was also defined as repeated statement and was specified with the subject cow. For samples collected three times (week 0, 6 and 11) during the trial week 0 was considered as covariate in the class statements. The covariate was used to make sure possible effects would not be observed only because of group differences already noted in week 0 (the first sampling time point). Best fitting covariance structures and models were tested using the Akaike information structures for a finite sample size (AICC). Effects were considered significant at probabilities *p* ≤ 0.05, while a trend was assumed for *p* ≤ 0.1. Before the variables SOD and GPx were evaluated with the statistical model the measurements were converted to hemoglobin in the erylysate. Results are presented as least square means and pooled standard error of means (**PSEM**). Correlation coefficient between different traits was estimated with Statistica 13.0 (StatSoft Inc., Tulsa, OK, USA) using Pearson correlation coefficient at *p* ≤ 0.01.

## 3. Results

### 3.1. Animal Performance

Table 2 shows milk production, body weight changes (BW) and DMI of both groups during the trial. Significant Group × Time (G × T) interactions were documented for all variables. 

The DMI (kg) of the CG was nearly constant over the whole trial, whereas the PG showed a decrease in total DMI (week 2 to 5: pasture plus TMR, week 5 to 11: pasture plus 4.5 kg concentrate per cow). During fulltime grazing the calculated DMI was on average 5 kg lower compared to CG, resulting in the Group × Time interaction. 

The milk performance (kg) of the PG decreased by 2 kg until week 5 and by further 5 kg compared to the initial value until week 11. For the last three weeks the milk performance seemed to stabilize.

In the CG an increase in body weight of a total of 34 kg compared to the initial value could be observed at the end of the trial. For the PG between weeks 0 to 6 a decrease in body weight (BW) of 34 kg compared to the initial value was observed, followed by an increase not reaching the initial value again.

### 3.2. Hematology

**Figure 1:**
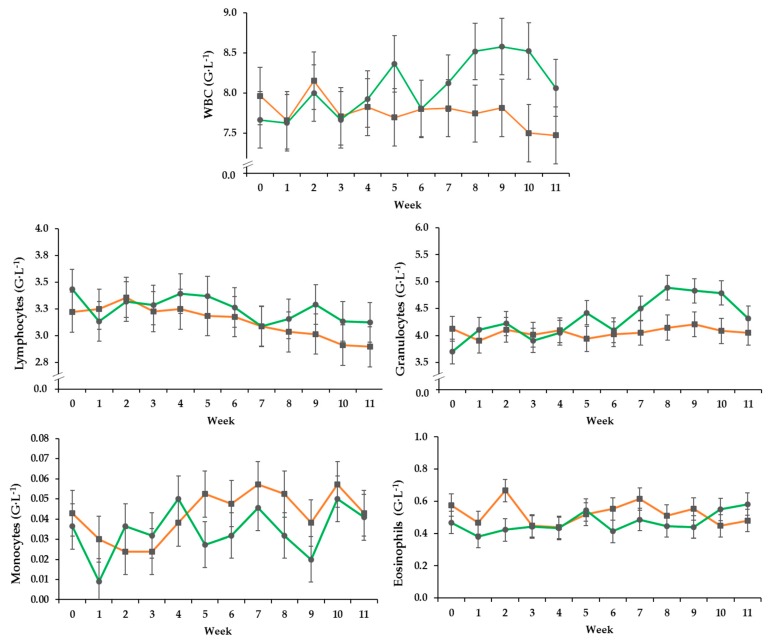
Effect of a ration change from an indoor based TMR to pasture on development of total white blood cell count, and numbers of granulocytes, eosinophils, lymphocytes and monocytes (G·L^−1^ = 10^9^ cells per Liter), all data points include standard error bars; • = pasture group (PG; *n* = 22, green); ■ = confinement group (CG; *n* = 21, orange). The results of the statistical analyses are shown in the accompanying table. The CG stayed on a TMR-based diet during the entire trial, while the PG was slowly introduced to a pasture-based ration: weeks 0 and 1 = TMR, week 2 = TMR and 3 h pasture·day^−1^, weeks 3 and 4 = TMR and 12 h pasture·day^−1^, week 5 to 11 = pasture and 4.5 kg of DM concentrate·day^−1^.

Total white blood cell and granulocyte counts of the PG increased over time whereas those of the CG fluctuated around the initial level, resulting in an effect by treatment over time. (Figure 1, Table 3). Lymphocyte counts decreased over time irrespective of treatment. Monocyte counts changed over time independent of group, while eosinophil counts differed between the groups independent of time resulting in a trend effect between the groups.

### 3.3. Serum Metabolites

The concentration of the acute phase protein haptoglobin resulted in a trend effect between the groups over the time. The trend appeared because of a short time increase of the haptoglobin concentration of the PG in the middle of the trial and a pronounced decrease at the end, whereas the concentration of the CG showed a more or less steady concentration (Table 4). 

The concentration of **Trp** increased over time in the CG, whereas the **Trp** concentration of the PG resulted in a decrease in the middle of the trial, followed by a pronounced increase. Its degradation product Kyn increased in both groups at all investigated time points (Table 4). The Trp/Kyn ratio was influenced by a single time effect, whereas animal treatment as well as the interaction of time and animal treatment had no effect on the ratio.

### 3.4. Flow Cytometry

#### 3.4.1. T-Cell Phenotyping

The percentage of CD4^+^ cells increased over time in both groups, whereby the elevation was more pronounced in the CG (Table 5), resulting in a group by time interaction. The percentage of CD8^+^ cells increased in the middle of the trial, and returned to start values afterwards in the PG, whereas the concentration in the CG increased at the end of the experiment. Therefore, the ratio of CD4^+^/CD8^+^ changed significantly over time independent of treatment group. The absolute numbers of CD4^+^ and CD8^+^ are determined by calculating the percentage with the lymphocyte count (blood count analysis, Celltaq). The increase of total CD4^+^ and CD8^+^ cell numbers showed only an effect over time without any influences of treatment which matches the results of the lymphocyte and monocyte cell counts.

#### 3.4.2. DHR Assay

**Figure 2:**
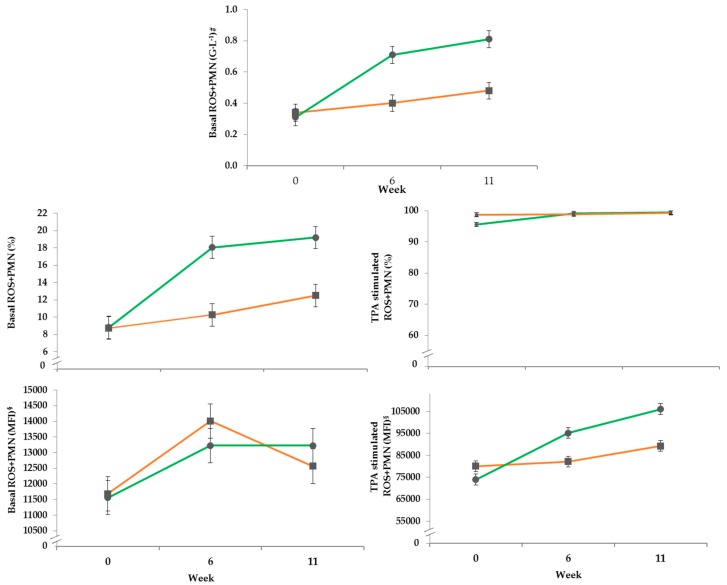
Effects of a ration change from an indoor based TMR to pasture on mean basal concentration (G·L^−1^ = 10^9^ cells per liter) and proportion (%) as well as basal and stimulated mean fluorescence intensity (MFI) of polymorph nuclear leucocytes; all data points include standard error bars; • = pasture group (PG; *n* = 22, green); ■ = confinement group (CG; *n* = 21, orange). The results of the statistical analyses are shown in the accompanying table. The CG stayed on a TMR-based diet during the entire trial, while the PG was slowly introduced to a pasture-based ration: weeks 0 and 1 = TMR, week 2 = TMR and 3 h pasture·day^−1^, weeks 3 and 4 = TMR and 12 h pasture·day^−1^, week 5 to 11 = pasture and 4.5 kg of DM concentrate·day^−1^.

In the course of the experiment the basal percentage of ROS produced by PMN increased to approximately 18% in the PG, while the CG reached only 10% after both had started from a similar level approximately 8%. The parameter was differentially affected by treatment over time (Table 6). The total granulocyte counts of ROS^+^PMN (G·L^−1^) in the PG was more altered by transition to pasture during the experiment compared to the control group in confinement (Figure 2, Table 6). The basal mean fluorescence intensity (MFI) of ROS^+^PMN was not affected by the experimental treatment, only time had a significant influence. Stimulation with TPA showed for the MFI of ROS^+^PMN a significant effect by treatment over time. The percentage of TPA-stimulated ROS^+^PMN stayed constant in the CG, whereas the first sampling of the PG at the beginning of the trial was lower compared to the other sampling timepoints. This increase led to an effect by treatment over time. After stimulation with TPA the MFI of ROS^+^PMN increased constantly from week 0 to 11 in both groups, but it was more pronounced in the PG resulting in an effect of treatment over time.

### 3.5. Enzyme Assay and Serum Metabolites

Red blood cell lysate concentration of hemoglobin (HGB in EL) showed a pronounced increase in the PG, whereas the RBC lysate concentration in samples of the animals belonging to the CG decreased over time resulting in a significant group by time interaction.

SOD activity in red blood cell lysate of the CG showed an increase over time, while the activity of the PG in the middle of the trial decreased and afterwards increased again resulting in a trend of treatment over time (Table 7). The GPx value of the CG increased first, followed by a decrease, whereas the PG showed no change resulting in a group by time interaction. 

A significant increase up to 127% of serum vitamin E concentration were demonstrated in the PG from week 0 to 11 (Table 7), resulting in an effect of group over time. The CG showed a significant decrease within the first six weeks, followed by a recovery. The calculated ratio of vitamin E to cholesterol in blood serum showed a more pronounced increase in the PG resulting in treatment differences over time, whereas the ratio of the CG fluctuated around the starting value. A pronounced increase of 25CHO was observed during transition to pasture within the PG and a strong decrease in the CG by the end of the experiment. For the serum 25ERG a more pronounced increase in the CG and a strong decrease in the PG were observed, causing a significant group by time interaction. The highest concentrations were noted in week 0 and 6 in the PG and in week 6 and 11 in the CG.

## 4. Discussion

Recently, we reported about the effects of a moderate concentrate feed supply (4.5 kg DM cow·day^−1^) after transition from an indoor based TMR to a full grazing ration with concentrate supply on the health and performance of mid lactation dairy cows [1]. In our experiment it was observed, that the red blood cell count and hematocrit changed significantly, while cholesterol, triglyceride, albumin and total protein concentrations remained uninfluenced [1]. Blood glucose homeostasis of both groups was not disturbed. Short time increase of blood NEFA and BHB as well as lipomobilization of fat depots and loss of BW demonstrated an energy lack of the PG during transition to pasture paralleled by a negative energy balance [1]. During the experimental time, rumen variables, for example fermentation pattern throughout the day, proportion of fatty acids and total volatile fatty acid concentration in rumen liquid changed in the PG, too. Continuously measured rumen pH and LPS concentrations did not reveal any increased risk for subacute ruminal acidosis (**SARA**) at any time and stage during the measurement period [2]. The transition time to pasture can be characterized and explored at different levels such as metabolic variables, behavior adaptions or rumen variables. Based on these data, the present experiment aimed at describing some parameters related to the immune system of dairy cows during transition to a full grazing ration with concentrate supply in comparison to cows housed in confinement. Circumstances with negative energy balance are well-known from calving and mostly associated with challenges of the immune system and relating blood parameters [9].

Regarding the hemogram, the number of WBC and granulocytes of the PG were obviously influenced by treatment, which is mirrored in marked alterations in the course of the experiment. It needs to be stressed that during the trial both groups did not show any serious symptoms/clinical signs of infectious diseases. Furthermore, for the PG no significant increase of eosinophil concentrations was detected for the PG during the measurement period; therefore, a pasturing-associated infestation and immune stimulation by parasites probably did not occur. The eosinophilic granulocytes of adult cattle are assumed to be 0.6 G·L^−1^ (0.3 to 0.9 G·L^−1^) under physiological conditions [34]. During the whole experiment both groups were within this range. It is difficult to assign the observed effects of increasing WBC and granulocytes in the PG to specific effects referring to the metabolism, especially as we know that the PG experienced lipomobilization and a negative energy balance due to higher physical activity and lower dry matter intake [1]. When assuming that the increase in granulocyte counts was driven by a subclinical infection, another marker of inflammation should have been influenced as well. Serum haptoglobin as the major bovine acute phase protein [35,36] showed in both groups pronounced fluctuations and high variation but at any time without significant differences between the groups. Haptoglobin concentrations measured during the trial were on average below a critical value of 250–10,000 mg·L^−1^, normally observed during SARA, clinical mastitis or experimentally induced aseptic inflammation [37]. This observation is in line with our documentation of no increased risk for SARA during transition to pasture [2]. On the other hand, the CG experienced a higher risk for SARA [2], evaluated by the SARA range of Zebeli et al. [38] (314 min·day^−1^ pH < 5.8 and daily average pH of 6.14), without critical changes in haptoglobin concentration. Saremi et al. [39] reported that haptoglobin is not only expressed by hepatocytes but can also be classified as a bovine adipokine. Therefore, changes in depot fat sizes might influence blood haptoglobin levels. In our trial we also observed a decrease in body fat of the PG and an increase for the CG [1] which could explain the short time increase in haptoglobin levels in the middle of the trial in the PG. As also reported by Saremi et al. [39], changes in serum haptoglobin concentrations can be connected to animal age and parity, too. The parity in our trial also differed between the animals within the same group which might additionally explain a part of the variation in haptoglobin levels in blood. The tryptophan/kynurenine (**Trp/Kyn**) ratio is another marker indicative of inflammation and provides indirect information about the activity of the enzyme indoleamine 2,3-dioxygenase [40,41,42]. During the period of calving, the activity of this enzyme can be reflected by a peak of Trp/Kyn ratio [6]. In this trial the Trp/Kyn ratio did not show any striking values, neither in the PG nor in the CG. However, the development of the documented inflammatory markers do not give any indication on present inflammatory processes during the experiment. The fluctuation of Trp could be ascribed to its role as a glycogenic- and ketogenic amino acid, producing intermediates for the citric cycle. However, the nearly uninfluenced concentrations of T-helper cells, of cytotoxic T-cells and their ratio support the view that T-cell homeostasis was not disturbed by treatment during the measured period. 

As already reported, the PG showed an energy loss during transition to pasture. Blood metabolites which relate to lack of nutrients, tissue mobilization or negative energy balance are known to reduce neutrophil function [10,11,43]. In our experiment BHB concentrations correlated positively with the percentage and MFI of unstimulated ROS^+^PMN (r = 0.225, *p* < 0.013; r = 0.2073, *p* < 0.021) as well as with ROS^+^PMN after stimulation (MFI; r = 0.2426, *p* < 0.007), whereas the NEFA concentration correlated negatively with the proportion of ROS^+^PMN after TPA stimulation (r = −0.1869, *p* < 0.038). It has to be mentioned, that the research of Scalia et al. [11] who performed in vitro experiments with NEFA concentrations up to 2 mmol·L^−1^ resulted in a compromised oxidative burst capacity and viability of PMNs. In our experiment the highest NEFA concentration was 0.45 ± 0.02 mmol·L^−1^ (LSmean ± PSEM; [1]). Nevertheless, in the present study influences on the immune system in terms of granulocytes concentration and modified cell function in case of ROS production could be observed during adaption to pasture. During transition to pasture and all day grazing with concentrate supply the PG exhibited a higher proportion and concentration of basal ROS^+^PMNs compared to the CG, whereas in both groups the capacity of basal ROS-formation in these cells seemed to be unchanged. After stimulation of oxidative burst by TPA, the capacity to produce free radicals (characterized by MFI) in PMN increased in the middle of the trial (week 6) approximately by 26% compared to week 0 and further on by 11% from week 6 to 11. The increase in PMN without any indication of infection diseases could improve the ability to fight against infections, as shown in human medicine [44]. In human experimental studies special methods are used to increase PMN numbers in cancer patients in order to improve their abilities to fight against infections [44]. These findings suggest that under certain conditions and despite of species differences high PMN concentrations could be beneficial for cows as they might also improve their ability to ward off clinical diseases for example in the periparturient period. Mainly in vitro studies in human neutrophils demonstrated that different physiological and microbial agents, like some chemoattractant cytokine or Toll-like receptors agonists, may prime ROS-production which means ‘preparing the enzyme NADPH-oxidase for a stronger activation’. The priming agents by themselves only induced a weak or no response, but after activation an enhanced ROS production was found [45]. The consequences of these results can only in an immune challenge model be investigated in more detail.

On the other hand, diet change from TMR to pasture and its effect on the gut can be another influencing factor of immune activity. Celi et al. [46] defined gut health as ‘a steady state where microbiome and the intestinal tract exist in symbiotic equilibrium and where the welfare and performance of the animal is not constrained by intestinal dysfunction’. Feeding change to high fermentable grass also requires a gradual diet adaption in order to allow rumen micro-organisms and their host to adjust to the substrate change [5,47]. The gastrointestinal tract for example is involved in digestion and nutrient absorption while representing an important component of the body’s immune system and can be out of balance through diet change resulting in immune modulation. Schären et al. [4] documented effects on rumen fermentation like lower pH values during transition to high fermentable grass. The authors concluded that the digestive system and its host need several weeks to adapt to the new feeding system. Gut health can be influenced by diet, microflora, immune system and intestinal integrity [12,13,46,47]. It plays a major role in dairy farming and is essential for production [46,47,48].

To sum up, we observed a pronounced increase of the proportion, absolute value and activity status of neutrophil cells, which might be interpreted as priming. We can only speculate that the priming was driven by physiological molecules like adiponectin due to reduction in bodyweight during transition to pasture or by microbial agents like lipopolysaccharides.

The fact that the animals as well as the immune system adapted without hints on oxidative stress (OS) could be shown in the development of the endogenous antioxidants variables. GPx and SOD are examples for endogenous antioxidants [49]. Bernabucci et al. [50] reported that plasma GPx activity could be an indirect indicator of OS during parturition. Oxidative stress is the result of an imbalance of oxidants and antioxidants [18]. We had expected, especially for the PG, an increase of the announced antioxidants, because of changing conditions of environment and metabolism and therefore situations connected with stress. In both groups, an increase of SOD could be documented over time, whereas in the PG the GPx value did not change during the trial. The short time increase of GPx and basal ROS-formation might be an indication of emotional stress in the CG in the middle of the trial when the PG left the stable. An example for an exogenous antioxidants is vitamin E [50]. Animals grazing on good quality pasture exhibit elevated concentration of that vitamin. This can be explained by higher concentrations of vitamin E in fresh green leaf tissue compared to silo feed. [51,52]. Politis et al. [53] examined the effects of vitamin E supplementation on isolated neutrophil function during parturition. They concluded that vitamin E supplementation (3000 IU/cow pre-partum and 1000 IU/cow post-partum) had positive effects on the function (superoxide production) of bovine neutrophils. Allison and Laven [54] observed that supplementation of high levels of vitamin E (at least 1000 IU per day) during the dry period and early lactation can reduce the incidence of mastitis, possibly because of an increase in immune system activity and function. The elevated concentration of vitamin E in the PG and its circulating form in the body defined by the ratio of vitamin E to cholesterol might have positive effects on the immune system [55] or rather is part of the body’s intracellular defense against the adverse effects of reactive oxygen species and free radicals [49,56]. These statements can be confirmed by a positive correlation between blood vitamin E concentration and proportion of unstimulated ROS^+^PMN (*r* = 0.201, *p* = 0.023). This could be an indication that vitamin E itself has a pro-oxidative effect. However, it needs to be stated that the increase in blood vitamin E concentration was not caused by pasture intake alone as the mineral mix also included vitamin E (described in experimental design and treatment).

During the transition period the vitamin D metabolite concentration of 25CHO of the PG increased significantly, whereas the concentration of the CG steadily decreased. The vitamin D metabolite 25ERG showed a decrease in the PG and the concentration of the CG fluctuated around the starting value. As reported in Descalzo et al. [57] 25ERG is mostly consumed from plant sources and 25CHO can be produced endogenously during sunlight exposure and further metabolic pathways. 25CHO is transported from skin through blood with the help of vitamin D binding proteins [58]. The endogenous synthesis of 25CHO was probably limited in the CG through indoor housing and therefore missing sunlight exposure. Between week 2 and 5 the average duration of sunshine was 9 h and during fulltime grazing 7 h which obviously enhanced 25CHO concentration in the PG. The decrease of sunlight exposure during the trial was influenced by weather conditions. The binding affinity of 25CHO to the vitamin D-binding protein is higher compared to 25ERG, documented by Hymøller and Jensen [59] in plasma samples. However, this could give a hint on why the concentrations of 25CHO were higher compared to 25ERG in serum samples. The influence of 25CHO on the animal can be diverse (intestinal calcium absorption and reabsorption from the bones and kidneys [60]. Waters et al. [61] showed that 25CHO regulates a few immune functions, mostly down-regulations of IFN-γ and upregulations of nitric-oxide production of peripheral blood mononuclear cells (PBMC) in vitro. In general, effects of vitamin D and its metabolites can be diverse and vary from alterations of circulating leucocyte numbers or modulating immune system [20,62], as observed in the present investigations. However, the impact is always dependent on the blood concentration [20]. Nelson et al. [63] reviewed that 25CHO concentrations higher than 30 ng·mL^−1^ influenced immune function in a positive manner. Hence, we cannot disregard the additional influence of sunlight on the different immune parameters, which can also be seen in a positive correlation of 25CHO and unstimulated ROS^+^PMN (%) as well as stimulated ROS^+^PMN (MFI) (r = 0.4408, *p* < 0.001; r = 0.1883, *p* = 0.037).

## 5. Conclusions

The results of the described trial show that the change of the management system in springtime can influence different blood parameters. Transition from confinement housing with TMR feeding to pasture plus moderate concentrate feed supply of 4.5 kg DM·day^−1^ was associated with an increase in white blood cell count, especially increasing granulocyte concentration and modified proportions of unstimulated ROS producing PMN. The changes in basal ROS production can be interpreted as an adaption of the PG to the new situation. Whether the improved vitamin E-status in PG is related to the observed effects in granulocytes needs to be clarified further. The observed effects are physiological responses of the animals during transition from confinement to pasture. Therefore, this period should be recognized as an immunologically challenging situation.

## Figures and Tables

**Table 1:** Chemical composition of experimental diets (mean ± SD).

Type of Feeding	Item ^2^ (g·kg^−1^ of DM, Unless Otherwise Noted)											
	DM (g·kg^−1^)	Ash	CP	uCP	NEL *	Sugar	Starch	RNB	CF	NDF	ADF	EE
**TMR ^1^ (CG/PG)**	471 ± 6	66 ± 0	158 ± 1	152 ± 0.5	6.8 ± 0	18 ± 0	253 ± 2.5	1.1 ± 0.3	184 ± 0	363 ± 0	205 ± 1.5	37 ± 0.5
**Pasture (PG)**	174 ± 23	94 ± 9	188 ± 19	142 ± 7	6.2 ± 0.3	114 ± 45	--	6.2 ± 1.9	231 ± 34	471 ± 40	254 ± 37	41 ± 5.8
**Concentrate (PG)**	899	108	93	148	7.6	27	579	8.9	31.5	124	42	30

^1^ TMR = total mixed ration; CG = confinement group; PG = pasture group. ^2^DM = dry matter; uCP = utilizable crude protein at the duodenum predicted by a regression of digested organic matter and undegraded feed protein as predicting variables. * NEL = net energy lactation (MJ·kg^−1^ of DM); RNB = ruminal nitrogen balance (=uCP − CP)/6.25; CF = crude fiber. NDF = neutral detergent fiber; ADF = acid detergent fiber; EE = ether extract.

**Table 2:** Effect of a ration change from total mixed ration (TMR) to pasture combined with concentrate supply on animal performance.

Variable ^1^	Group ^2^	Week												PSEM ^3^	*p*-Value		
		0	1	2	3	4	5	6	7	8	9	10	11		G	T	GxT
**DMI (kg/d) ^4^**	CG	20.5	21.1	22.2	21.5	22.4	22.4	23.0	21.8	21.7	23.4	22.3	20.8	0.9	<0.001	<0.001	<0.001
**DMI (kg/d) ^5^**	PG	20.3	20.7	20.1	19.6	18.5	17.8	18.4	16.3	18.4	16.1	17.5	17.2				
**Milk yield**	CG	29.3	28.3	27.5	28.8	28.9	28.9	28.6	28.1	28.4	28.0	27.3	27.2	2.7	0.013	<0.001	<0.001
**(kg/d)**	PG	29.1	29.4	29.8	29.4	26.0	27.1	25.5	25.1	24.7	22.6	23.8	22.8				
**Body weight**	CG	620	623	624	621	621	627	627	633	636	645 ^6^	653	654	13	0.079	<0.001	<0.001
**(kg)**	PG	620	619	607	600	593	588	578	581	584	597 ^6^	610	612				
**NEB (MJ NEL·day^−1^)**	CG	−5.5	−2.0	6.0	0.4	5.5	6.8	12.8	4.9	2.1	13.4	6.8	−3.5	2.3	0.003	<0.001	<0.001
	PG	−2.2	−2.1	−1.0	−4.2	−3.9	−3.6	−3.7	−4.3	−4.1	−3.9	−4.3	−4.1				

^1^ Dry matter intake (DMI), net energy balance (NEB; mega joule net energy for lactation per day; calculated from the difference of energy intake minus energy for requirements for maintenance minus energy requirements for performance minus energy requirements for gestation); Material and Methods are described in detail in Hartwiger et al. [1]. ^2^ The CG stayed on a TMR-based diet during the entire trial, while the PG was slowly introduced to a pasture-based ration: weeks 0 and 1 = TMR, week 2 = TMR and 3 h pasture·day·^−1^, weeks 3 and 4 = TMR and 12 h pasture·day·^−1^, week 5 to 11 = pasture and 4.5 kg DM concentrate·cow·^−1^·day·^−1^. ^3^ PSEM = pooled standard error of the mean; G = group, T = time. ^4^ DMI was documented by means of weighing troughs. ^5^ DMI in weeks 0 and 1 was documented by means of weighing troughs, DMI in week 2 to 5 was documented by means of weighing troughs plus calculated DMI on pasture (method described in Hartwiger et al. [1]), DMI on pasture in week 5 to 11 was calculated as described, including 4.5 kg DM concentrate·cow·^−^^1^·day^−1^. ^6^ Due to technical problems the BW of week 9 had to be assumed as the mean of weeks 8 and 10.

**Table 3:** Results of the statistical analyses.

Variable	PSEM ^1^	*p*-Value		
		Group (G)	Time (T)	G × T
WBC (G·L^−1^) *	0.34	0.554	0.038	0.002
**Lymphocytes (G·L^−1^)**	0.19	0.674	<0.001	0.249
**Granulocytes (G·L^−1^)**	0.20	0.345	0.002	0.012
**Monocytes (G·L^−1^)**	0.01	0.163	0.021	0.710
**Eosinophils (G·L^−1^)**	0.07	0.082	0.595	0.683

^1^ PSEM: Pooled standard error of the mean. * G·L^−1^ = 10^9^ cells per Liter.

**Table 4:** Effect of a ration change from TMR to pasture on blood variables: haptoglobin, tryptophan (Trp) and kynurenine (Kyn).

Variable ^1^	Group ^2^	0	6	11	PSEM ^3^	*p*-Value		
						Group (G)	Time (T)	G × T
**Haptoglobin ^4^ (μg·mL^−1^)**	CG	204	108	239	136	0.602	0.820	0.058
	PG	192	482	92				
**Tryptophan (Trp)** **(mg·L^−1^)**	CG	5.7	5.8	6.4	0.19	0.571	<0.001	0.186
	PG	5.6	5.2	6.1				
**Kynurenine (Kyn)** **(mg·L^−1^)**	CG	1.05	1.10	1.24	0.05	0.208	<0.001	0.422
	PG	0.95	0.93	1.29				
**Trp/Kyn**	CG	0.19	0.18	0.20	0.01	0.391	0.012	0.434
	PG	0.24	0.19	0.20				

^1^ Week 0 was set as covariate, integrated in the MIXED procedure of SAS with diet group and week as fixed factors. ^2^ The CG stayed on a TMR-based diet during the entire trial, while the PG was slowly introduced to a pasture-based ration: weeks 0 and 1 = TMR, week 2 = TMR and 3 h pasture·day^−1^, weeks 3 and 4 = TMR and 12 h pasture·day^−1^, week 5 to 11 = pasture and 4.5 kg of DM concentrate·day^−1^. ^3^ PSEM = pooled standard error of the mean. ^4^ Upper tolerance limit: 250 μg·mL^−1^.

**Table 5:** Effects of a ration change from an indoor based TMR to pasture on cell numbers and proportions of CD4^+^ and CD8^+^ cells relative to total mononuclear cells.

Variable ^1^	Group ^2^	Week			PSEM ^3^	Group (G)	Time (T)	*p*-Value
		0	6	11				G × T
CD4^+^ (G·L^−1^) *	CG	0.77	0.86	0.79	0.02	0.052	0.005	0.129
	PG	0.78	0.79	0.76				
**CD4^+^ (%)**	CG	23.4	26.7	27.1	0.43	0.009	<0.001	0.008
	PG	23.7	25.2	24.9				
CD8^+^ (G·L^−1^) *	CG	0.35	0.41	0.36	0.07	0.032	0.000	0.245
	PG	0.35	0.37	0.34				
**CD8^+^ (%)**	CG	10.8	12.7	12.2	0.21	0.002	<0.001	0.008
	PG	10.7	11.9	10.9				
**CD4^+^/CD8^+^**	CG	2.31	2.26	2.35	0.05	0.407	0.001	0.455
	PG	2.32	2.24	2.47				

^1^ CD4^+^ = T-helper cells; CD8^+^ = cytotoxic T-cells; week 0 was set as covariate, integrated in the MIXED procedure of SAS with diet group and week as fixed factors. ^2^ CG = confinement group (*n* = 21); PG = pasture group (*n* = 22). The CG stayed on a TMR-based diet during the entire trial, while the PG was slowly introduced to a pasture-based ration: weeks 0 and 1 = TMR, week 2 = TMR and 3 h pasture·day^−1^, weeks 3 and 4 = TMR and 12 h pasture·day^−1^, week 5 to 11 = pasture and 4.5 kg of DM concentrate·day^−1^. ^3^ PSEM = pooled standard error of the mean. * CD4^+^ or rather CD8^+^ = (Lymphocyte count (G·L^−1^) + Monocyte count (G·L^−1^)) / CD4^+^ (%) or rather CD8^+^ (%); G·L^−1^ = 10^9^ cells per Liter.

**Table 6:** Results of the statistical analyses.

Variable ^1^	PSEM ^2^	*p*-Value		
		Group (G)	Time (T)	G × T
*Basal unstimulated*				
**ROS^+^PMN (G·L^−1^) ^3^**	0.05	<0.001	<0.001	<0.001
**ROS^+^PMN (%)**	1.10	<0.001	<0.001	0.001
**ROS^+^PMN (MFI) ^4^**	546	0.873	0.001	0.363
*TPA stimulated*				
**ROS^+^PMN (%)**	0.67	0.399	<0.001	0.003
**ROS^+^PMN (MFI)**	2437	0.002	<0.001	<0.001

^1^ ROS = reactive oxygen species; PMN = polymorph nuclear cells; MFI = mean fluorescence intensity; TPA = tetradecanoyl-12, 13-phorbol acetate; week 0 was set as covariate, integrated in the MIXED procedure of SAS with diet group and week as fixed factors. ^2^ PSEM: Pooled standard error of the mean. ^3^ Peripheral blood granulocytes exhibiting a basal ROS production (total blood granulocytes + eosinophil, multiplied with the proportion of basal ROS^+^PMN) according to Dänicke et al. [32]; **G·L**^−1^ = 10^−9^ cells per Liter. ^4^ Arbitrary units.

**Table 7:** Effects of a ration change from an indoor based TMR to pasture on blood variables.

Variable ^1^	Group ^2^	0	6	11	PSEM ^3^	*p*-Value		
						Group (G)	Time (T)	G × T
**HGB in EL** **(g·dL^−1^)**	CG	2.63	2.48	2.33	0.09	0.006	0.372	0.016
	PG	2.51	2.82	2.70				
**SOD** **(mU·g Hb^−1^) ^#^**	CG	3.3	3.5	4.0	0.19	0.759	<0.001	0.099
	PG	3.6	3.1	4.4				
**GPx** **(mU·mg Hb^−1^) ^#^**	CG	225	280	218	8.8	0.964	0.001	0.002
	PG	237	242	242				
**Vitamin E (mg·L^−1^)**	CG	6.47	5.34	6.34	0.27	<0.001	<0.001	<0.001
	PG	6.48	8.18	8.79				
**Vitamin E/cholesterol**	CG	0.53	0.48	0.57	0.03	<0.05	<0.001	<0.001
	PG	0.50	0.59	0.68				
**25CHO (μg·L^−1^)**	CG	38.7	35.2	29.2	1.4	<0.001	<0.001	<0.001
	PG	36.8	52.6	61.0				
**25ERG (μg·L^−1^)**	CG	16.3	19.3	18.8	0.6	<0.001	<0.001	<0.001
	PG	15.3	16.1	10.8				

^1^ HGB in EL = hemoglobin in erythrocyte lysate; SOD = superoxide dismutase; GPx = glutathione peroxidase; 25CHO= 25-hydroxycholecalciferol (metabolite of Vit. D_3_); 25ERG = 25-hydroxyergocalciferol (metabolite of Vit. D_2_); week 0 was set as covariate, integrated in the MIXED procedure of SAS with diet group and week as fixed factors. ^2^ CG = confinement group (*n* = 21); PG = pasture group (*n* = 22). The CG stayed on a TMR-based diet during the entire trial, while the PG was slowly introduced to a pasture-based ration: weeks 0 and 1 = TMR, week 2 = TMR and 3 h pasture·day^−1^, weeks 3 and 4 = TMR and 12 h pasture·day^−1^, week 5 to 11 = pasture and 4.5 kg of DM concentrate·day^−1^. ^3^ PSEM = pooled standard error of the mean. ^#^ Referred to hemoglobin in erythrocyte lysate.
